# Determinants of curvature constant (W’) of the power duration relationship under normoxia and hypoxia: the effect of pre-exercise alkalosis

**DOI:** 10.1007/s00421-017-3574-4

**Published:** 2017-03-09

**Authors:** Sanjoy K. Deb, Lewis A. Gough, S. Andy Sparks, Lars R. McNaughton

**Affiliations:** Department of Sport and Physical Activity, Edge Hill University, St Helen’s Road, Ormskirk, L39 4QP UK

**Keywords:** Critical power, Sodium bicarbonate, Altitude, Severe intensity domain

## Abstract

**Purpose:**

This study investigated the effect of induced alkalosis on the curvature constant (W’) of the power-duration relationship under normoxic and hypoxic conditions.

**Methods:**

Eleven trained cyclists (mean ± SD) Age: 32 ± 7.2 years; body mass (bm): 77.0 ± 9.2 kg; VO_2peak_: 59.2 ± 6.8 ml·kg^−1^·min^−1^ completed seven laboratory visits which involved the determination of individual time to peak alkalosis following sodium bicarbonate (NaHCO_3_) ingestion, an environment specific ramp test (e.g. normoxia and hypoxia) and four x 3 min critical power (CP) tests under different experimental conditions. Participants completed four trials: alkalosis normoxia (ALN); placebo normoxia (PLN); alkalosis hypoxia (ALH); and placebo hypoxia (PLH). Pre-exercise administration of 0.3 g.kg^−1^ BM of NaHCO_3_ was used to induce alkalosis. Environmental conditions were set at either normobaric hypoxia (FiO_2_: 14.5%) or normoxia (FiO_2_: 20.93%).

**Results:**

An increase in W’ was observed with pre-exercise alkalosis under both normoxic (PLN: 15.1 ± 6.2 kJ vs. ALN: 17.4 ± 5.1 kJ; *P* = 0.006) and hypoxic conditions (ALN: 15.2 ± 4.9 kJ vs. ALN: 17.9 ± 5.2 kJ; *P* < 0.001). Pre-exercise alkalosis resulted in a larger reduction in bicarbonate ion (HCO_3_^−^) concentrations during exercise in both environmental conditions (*p* < 0.001) and a greater blood lactate accumulation under hypoxia (*P* = 0.012).

**Conclusion:**

Pre-exercise alkalosis substantially increased W’ and, therefore, may determine tolerance to exercise above CP under normoxic and hypoxic conditions. This may be due to NaHCO_3_ increasing HCO_3_^−^ buffering capacity to delay exercise-induced acidosis, which may, therefore, enhance anaerobic energy contribution.

## Introduction

Fatigue is determined by an array of factors that are suggested to originate from afferent/efferent feedback from the central nervous system (i.e. central fatigue), and/or metabolic and biochemical alterations in the intramuscular regions (i.e. peripheral fatigue) (Ament and Verkerke 2009). The mediators of fatigue are, however, predominantly dependant on exercise intensity and as such, the physiological response and tolerance to exercise can be clustered into distinct domains (Burnley and Jones 2007). Tolerance within the severe intensity domain can be described by a mathematical hyperbolic curve known as the power–duration relationship (Jones et al. 2010; Poole et al. 2016). This hyperbolic curve can be categorised into the asymptote and the curvature constant, which in the power–duration relationship are known as critical power (CP) and W’, respectively. The CP signifies the greatest intensity where a physiological steady state can be maintained (Poole et al. 1988); whilst also defining the boundary between heavy and severe exercise intensity domains (Burnley and Jones 2007). The range of exercise intensities between CP and the power output at peak oxygen uptake (VO_2peak_) represents the W’. This quantifies a fixed work constant [measured in kilojoules (kJ)] available for exercise within the severe exercise intensity domain (Jones et al. 2010; Poole et al. 2016) and, therefore, enables the prediction of time to exercise exhaustion at any given power output above CP. Exercise within the W’ range is associated with a distinct physiological response that are associated with fatigue (Poole et al. 1988; Burnley and Jones 2007; Jones et al. 2008). This includes, muscle metabolite perturbations, such as an exponential rise in muscle hydrogen cations (H^+^) and phosphocreatine (Pcr) breakdown (Jones et al. 2008); as well as the development of a substantial VO_2_ slow component (Murgatroyd et al. 2011).

The power–duration relationship has varying applications to healthy, patient and athletic populations. More specifically this includes; the normalisation and prescription of exercise intensities (Ferguson et al. 2013), a method to predict exercise performance capabilities (Black et al. 2014) and inform athletic pacing strategies (Vanhatalo et al. 2011). Furthermore, the critical power concept has been cited as a method that can assess the effect of interventions by providing both functional exercise capacity data and associated physiological benefits (Whipp and Ward 2009; Jones et al. 2010). Therefore, greater mechanistic appreciation of the physiological components that determine CP and W’ may improve the practical application of this power–duration model. Critical power is sensitive to manipulation in O_2_ availability with hypoxic exposure diminishing CP (Derkerle et al. 2012; Simpson et al. 2015) and hyperoxic exposure increasing CP (Vanhatalo et al. 2010a). As such CP is suggested to be predominantly comprised of oxygen dependant, aerobic energy sources (Poole et al. 2016). In comparison, the determinants of W’ are less clear (Jones et al. 2010; Poole et al. 2016) as interventions that increase CP can concurrently reduce W’ (Vanhatalo et al. 2010a). This may indicate that W’ may not only represent the finite anaerobic energy contribution, as originally proposed, but rather an interplay of aerobic and anaerobic energetic factors.

Muscle metabolite disturbances are closely linked with W’ (Jones et al. 2008) and the exogenous manipulation of metabolite disturbances can enhance W’, such as creatine ingestion to increase PCr resynthesise and thus W’ (Miura et al. 1999). This, however, has not been consistently demonstrated, as pre-exercise alkalosis (through NaHCO_3_ supplementation) was shown not to alter W’, despite the alleviation of H^+^ accumulation during all-out exercise (Vanhatalo et al. 2010b). Indeed, there is considerable debate regarding the involvement of acidosis in fatigue and exercise performance (Westerblad 2016; Fitts 2016), which may explain the lack of improvement reported in W’. Morales-Alamo et al. (2015) observed that sprint performance following exhaustive exercise recovered at a faster rate than muscular acidosis (i.e. removal of H^+^). This suggests a close temporal relationship between acidosis and whole body exercise performance may not coexist. Furthermore, performance recovery was faster with hypoxic exposure, with the authors suggesting central fatigue mechanisms may have a more prominent role in fatigue in comparison to normoxia. This is despite acute hypoxia eliciting an additive acidic stress during exercise (Hogan et al. 1983), which is identified as a factor contributing to the diminished exercise performance under this environmental stressor (Clark et al. 2007). While, NaHCO_3_ supplementation is shown to be an effective ergogenic aid by acting to delay the onset of acidosis during exercise (McNaughton et al. 2008, 2016), suggesting acid–base balance is implicated with exercise performance and fatigue. Furthermore, exercise above CP is associated with an increase in H^+^ and, therefore, the hypothesis identifying acidosis as a physiological determinant of W’ utilisation cannot be dismissed.

Theoretically, NaHCO_3_ may enhance W’ by increasing the availability of blood HCO_3_^−^ and strengthen the physiochemical buffering capacity, which acts to dampen the rate of H^+^ accumulation during exercise (Carr et al. 2011). More recent methodological developments in NaHCO_3_ administration suggest an inter-individual variability in extracellular peak blood alkalosis, which ranges from 10 to 140 min (Stannard et al. 2016; Miller et al. 2016). Consequently, previous research utilising standard ingestion times across all participants may not have induced individual peak alkalosis, such as that used by Vanhatalo et al. (2010b). Thereby limiting the potential to attain an accurate representation of the effect of alkalosis. The purpose of this study was, therefore, to investigate the effect of individualised pre-exercise NaHCO_3_ supplementation on CP and W’ during the 3 min critical power test under normoxic and hypoxic conditions.

## Methods

### Participants

Eleven male trained cyclists volunteered to participate in this study with the following mean ± SD physical characteristics, age 32 ± 7.2 years; body mass 77.0 ± 9.2 kg; VO_2peak_ 59.2 ± 7.4 ml·kg^−1^·min^−1^; peak power output 391.3 ± 43.7 W. Participant inclusion was determined by age (18–40 years), training history (minimum of 2 years cycling and a 7 h·week^−1^ minimum training volume) and previous altitude exposure (not resided at altitude for the previous 6 months). Written informed consent was obtained from all participants after explanation of test procedures and associated risks. Ethical approval was obtained for the study from the Departmental Research Ethics Committee and the study was conducted in accordance with the Declaration of Helsinki.

### Experimental overview

Participants visited the laboratory on seven separate days at the same time of day (±1 h), with visits separated by at least two, but no more than 7 days. Participants were instructed to refrain from strenuous exercise and alcohol 24 h prior to each trial; while participants abstained from caffeine on the day of testing. Dietary intake was recorded 24 h preceding the initial trial, which was replicated for subsequent visits and confirmed verbally on the commencement of each trial. Participants were instructed to arrive in a 3 h postprandial state and a euhydrated state by maintaining fluid intake prior to arrival to limit confounding nutritional effects on exercise performance.

During the first visit, individual time to peak blood alkalosis was established from the ingestion of 0.3 g·kg^−1^ body mass of NaHCO_3_ through mapping time course of blood response over 90 min (Miller et al. 2015). Capillary blood samples (70 µl) were taken prior to NaHCO_3_ ingestion followed by samples every 10 min for 60 min post-ingestion and then every 5 min from 60 to 90 min. Samples were collected in a seated position from a sterilised finger into a capillary tube (Electrolyte balanced heparin clinitube, Radiometer, Denmark). Arterialisation was attained using an electric heated blanket (45 °C) for 5 min prior to sample collection (Yang et al. 2012). Samples were analysed for blood pH (Radiometer ABL90 Flex, Denmark) to determine the time taken for peak pH to transpire. This formed the timescale for individualised NaHCO_3_ ingestion during subsequent experimental trials.

Exercise trials were all conducted within a normobaric environmental chamber (Model S016r-7-sp TISS, UK), with ambient temperature (21 °C) and humidity (40%) regulated throughout. Fractional inspired oxygen (FiO_2_%) was adjusted for normoxic (FiO_2_ = 20.93%) and hypoxic (FiO_2_ = 14.5%) environmental conditions, which are equivalent to sea level and 3000 m, respectively. Environmental conditions were single blind and randomised during experimentation. A ‘sham hypoxic’ condition was created during the normoxic trials to mask the environmental conditions. This involved maintaining the oxygen (O_2_) controls to replicate auditory cues between conditions, a procedure previously used by Gallagher et al. (2014). Participants entered the chamber 10 min prior to exercise to allow equilibrium between atmospheric and body O_2_ stores (Andreassen and Rees 2005).

The first two exercise trials consisted of an incremental ramp test assigned in a single-blinded, random order to either the normoxic or hypoxic environmental condition. A randomised, cross-over design was employed for the following four experimental trials under different environmental conditions. All exercise tests were performed to replicate the original ramp and 3 min all-out critical power test performed by Vanhatalo et al. (2007) to maintain the validity of the CP and W’ measured. The four experimental conditions were: alkalosis normoxia (ALN), placebo normoxia (PLN), alkalosis hypoxia (ALH), and placebo hypoxia (PLH). Supplements were administered in a double-blinded manner prior to exercise at the time to peak alkalosis identified in the initial trial. Participants ingested 0.3 g·kg^−1^ body mass of NaHCO_3_ or a placebo of sodium chloride (NaCl) at 0.21 g·kg^−1^ body mass, which was calculated as an equimolar sodium concentration to the NaHCO_3_ dose. Supplements were mixed with 400 ml of water and 50 ml of sugar-free orange-flavoured cordial, which were ingested within a 5-min period. Capillary blood samples were taken during experimental trials on three occasions (Pre-supplement, Pre-exercise and within 1 min Post-exercise) using the method described earlier. Samples were analysed for blood H^+^ concentrations ([H^+^]), bicarbonate ions concentration ([HCO_3_^−^]), blood lactate concentration ([bla]) and O_2_ saturation percentage (SpO_2_), blood oxygen (PO_2_) and carbon dioxide (PCO_2_) partial pressure (Radiometer ABL90 Flex, Denmark).

### Determination of VO_2peak_ and ventilatory threshold 1

All exercise trials were conducted using an electromagnetically braked cycle ergometer (Lode Excalibur Sport, Groningen, The Netherlands). Participants adjusted the ergometer seat and handle bars to maximise comfort, this was recorded in the first trial and replicated for all subsequent trials. The incremental ramp test was performed with the ergometer in isokinetic mode and was proceeded by a 5 min unloaded warm-up. The test commenced at 75 W for 1 min and followed by continuous increase of 1 W every 2 s (30 W·min^−1^). Participants selected a preferred cadence (80 rpm, *n* = 5; 90 rpm, *n* = 6) to maintain throughout the test until volitional exhaustion. This test was terminated when cadence fell by more than 10 rpm from the preferred cadence for more than 5 s, despite strong verbal encouragement.

Breath-by-breath pulmonary gas exchange and heart rate was measured throughout the incremental ramp test, and all subsequent trials, using a free standing metabolic gas analyser (K5, Cosmed, Italy). Calibration was performed prior to every test in accordance with manufacturer’s instructions. Pulmonary gas exchange data were initially smoothed, by 4 standard deviations from the mean, to remove errant data points caused by coughing or swallowing. Data were then averaged to 10 s bins for the identification of the first ventilatory threshold (VT1). The VT1 was detected through inflection points on gas exchange graphs using the following criteria: (1) the v-slope method through the breakpoint in VCO_2_/VO_2_ against time; (2) an increase in V_E_/VO_2_ but no increase in V_E_/VCO_2_; and (3) an increase in P_ET_O_2_ without a decline in P_ET_CO_2_ (Beaver et al. 1986). The subsequent power output associated with the VT1 was then used to determine the linear factor for the 3 min critical power test (as described below). Peak power output (PPO) was defined as the greatest power output attained at the termination of the test. Peak oxygen uptake was defined as the highest 5 s rolling average of VO_2_. Furthermore, cardiopulmonary data including peak V_E_ and RER; and average V_E_/VCO_2_, V_E_/VO_2_, P_ET_CO_2_ and P_ET_O_2_ were documented.

### Critical power test

The CP test consisted of an initial 3 min unloaded baseline pedalling at a preferred cadence followed by the commencement of the 3 min maximal sprint phase. On the sprint phase the ergometer was set on the linear mode which provides a fixed resistance. This linear factor (linear factor = power/cadence^2^) was calculated to determine a fixed resistance that results in the participants preferred cadence (used during the incremental ramp protocol) to be attained at a power output that corresponds to the mid-point of peak power output and the VT1 intensity. This linear factor was determined for the respective environmental condition to avoid substantially larger determinations of W’ under hypoxia (Simpson et al. 2015). During the last 10 s of unloaded pedalling, participants were asked to increase cadence to 120 rpm, and given a countdown into the commencement of the sprint phase. Participants were instructed to attain a peak power on sprint phase initiation and hold the cadence as high as possible throughout test duration. Strong verbal encouragement was provided by the same researcher throughout the study and information of cycling cadence was given during the test, whilst information related to performance and time elapsed was withheld, to avoid pacing. Familiarisation to the critical power test was conducted following a 30 min recovery after the second incremental ramp test, a design that has previously been shown to retain the validity of the 3 min CP test (Constantini et al. 2014). The determination of CP and W’ using the 3 min test has been subject to criticism due to the suggested overestimation of the CP parameter (Bergstrom et al. 2013); therefore, a minimal 30 s rolling average was taken as CP and the total work performed above CP as W’. This replicates the analysis previously used by Shearman et al. (2016) who found a lower intensity than the original CP determination method, thus limiting the risk of overestimation. In addition, the original identification of CP through last 30 s end power output, and the total work performed above this power output, was calculated for comparative purposes and defined as EP and W’_EP_. The PPO was determined as the greatest 5 s average power output recorded during the CP test. While, heart rate was recorded throughout the exercise test (T31, Polar, Finland) and participant were asked to rate perceived exercise (RPE) using the 6–20 point Borg scale at the end of exercise.

### Statistical analysis

The Shapiro–Wilk test provided no evidence to reject the hypothesis that all data were normally distributed. A two-way ANOVA [condition (normoxia vs. hypoxia) × time] was used to compare power outputs of VT1 and PPO during the incremental ramp test, whilst cardiopulmonary variables between environmental conditions were compared using a paired *t* test. A two-way [treatment (alkalosis vs. placebo) × condition (normoxia vs. hypoxia)] repeated measures ANOVA was used to compare means for dependant variables within the 3 min CP test (CP, W’, total work done (TWD), PPO, and HR) and RPE. A three-way [treatment (alkalosis vs. placebo) × condition (normoxia vs. hypoxia) × time (pre-supplementation vs. pre-exercise vs. post-exercise)] repeated measures ANOVA was conducted to compare blood [HCO_3_^−^] and [H^+^], PO_2_ and PCO_2_, whilst further comparison of change in blood [HCO_3_^−^], [H^+^] and lactate during exercise were conducted through a two-way ANOVA. Furthermore, a comparison of CP to EP and W’ to W’_EP_ was performed using a two-way ANOVA. Where significant main effects were found, a Bonferroni correction was used for post hoc pair-wise analyses. Effect size were calculated using partial eta squared (ηp^2^) and interpreted as small (<0.01), medium (0.01–0.06) and large effect (≥0.14) (Cohen 1988). Pearson correlations were performed to examine the relationship between the effect of NaHCO_3_ on TWD and the effect on W’ and CP, under both environmental conditions. A magnitude-based inferences approach was used to detect the likely practical outcome of the intervention (Batterham and Hopkins 2006). The smallest meaningful change was assessed against a Cohen unit of 0.2. This contemporary statistical inference method provides an interpretation on the magnitude of an effect against a pre-determined smallest meaningful effect from the treatment. This, therefore, substantiates inferences from null hypothesise significance testing and effect sizes, whilst also reducing inferential error rates (Hopkins and Batterham 2016). The following qualitative descriptors were applied to quantitative percentile scores: (1) 25–75% possible; (2) 75–95% likely; (3) 95–99% very likely and (3) > 99% most likely. Instances where the likelihood of a beneficial or negative effect were > 5%, the qualitative interpretation was deemed to be unclear. Descriptive data are presented as mean ± SD and statistical significance accepted at *p* < 0.05. Data were analysed using SPSS v22 for Windows (SPSS Inc., Chicago, IL, USA), except for magnitude based inferences which were calculated through an online spreadsheet (Batterham and Hopkins 2006).

## Results

During the preliminary trial, time to peak pH following 0.3 g·kg^−1^ bm of NaHCO_3_ ingestion ranged between 40 and 70 min (mean: 50 ± 9.6 min). Ingestion of NaHCO_3_ during subsequent trials demonstrated a good correlation with the individualised dose response trial with an ICC of 0.6; and a standard error of measurement as 0.007 and 0.006 for trials ALN and ALH, respectively. A significant interaction between environment was found (*p* = 0.005; ηp^2^ = 0.57) on peak power during the ramp test (Table 1). This was represented in peak power output reducing by 10% (*p* < 0.001) between normoxia to hypoxia; although power output at VT1 was similar between environmental conditions (*p* = 0.38). There was also a significant 18% reduction in VO_2peak_ between normoxic to hypoxic environment (*p* = 0.001).

**Table 1:** RAMP test result under normoxic and hypoxic conditions

Variables	Normoxia	Hypoxia
VO_2_ max (ml.kg−1.min−1)	59.3 ± 7.4*	48.5 ± 6.0
PPO (W)	390.4 ± 45.0*	351.5 ± 40.1
VT1power (W)	176.36 ± 18.17	166.8 ± 19.4
VEpeak	151.2 ± 34.2	151.3 ± 35.3
RER	1.2 ± 0.1	1.3 ± 0.2
VE/VCO_2_	24.6 ± 5.5*	31.4 ± 6.8
VE/VO_2_	24.5 ± 2.8*	28.6 ± 4.4
PETO_2_ (mm Hg)	87.1 ± 25.7*	60.8 ± 14.0
PETCO_2_ (mm Hg)	47.3 ± 3.9*	35.0 ± 6.7
HRpeak (bpm)	181 ± 6.6	179 ± 7.1

Values reported as a mean ± SD

*Denotes significantly different to Hypoxia (*p* < 0.05)

**Fig. 1:**
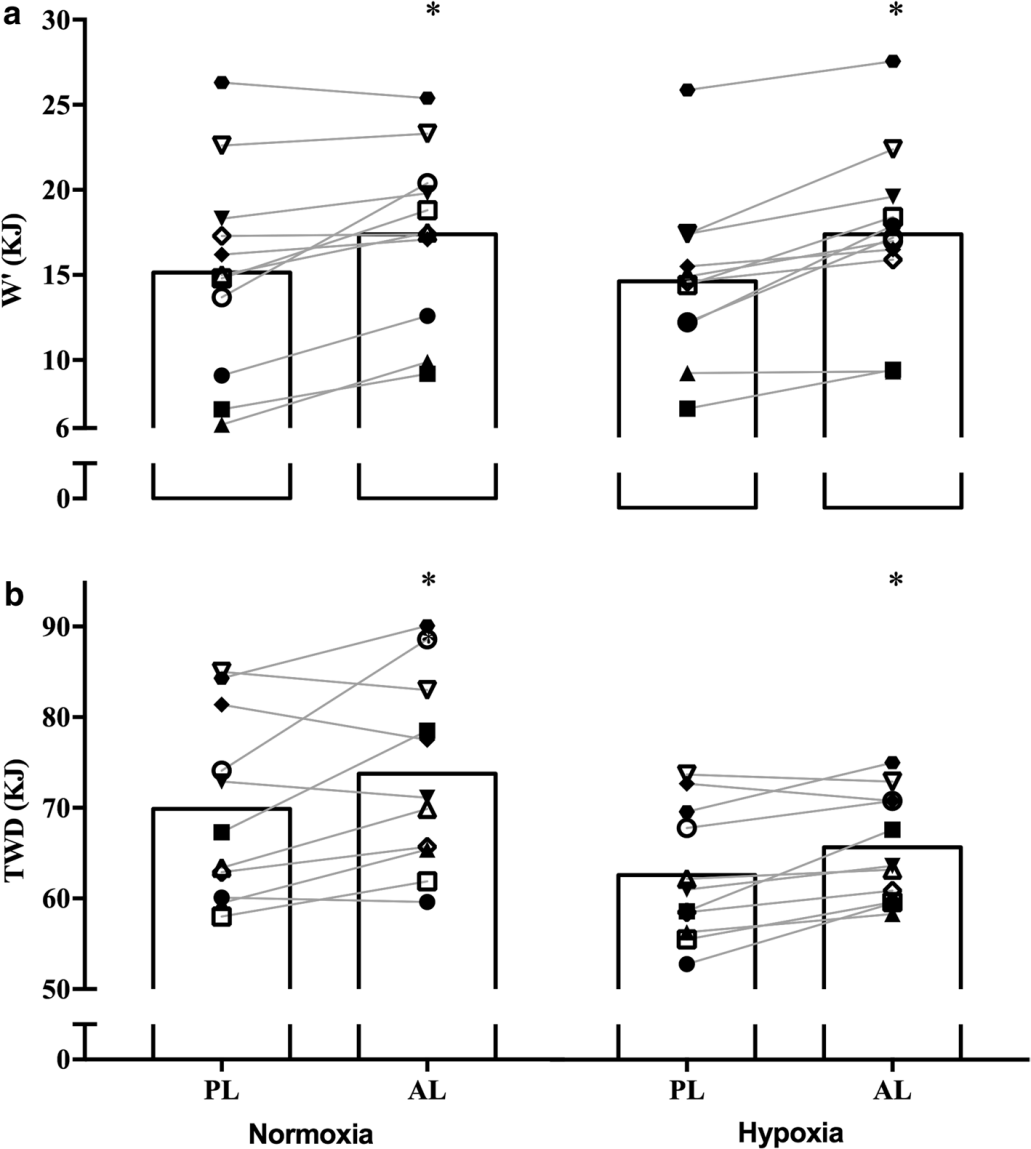
Graph **a** displays W’ and graph **b** displays TWD. These figures represent the mean values in each experimental condition, while the individual points and connecting lines represent the individual response to alkalosis under the receptive environmental conditions. *Asterisk* represents significantly different to placebo condition (*p* < 0.05)

**Fig. 2:**
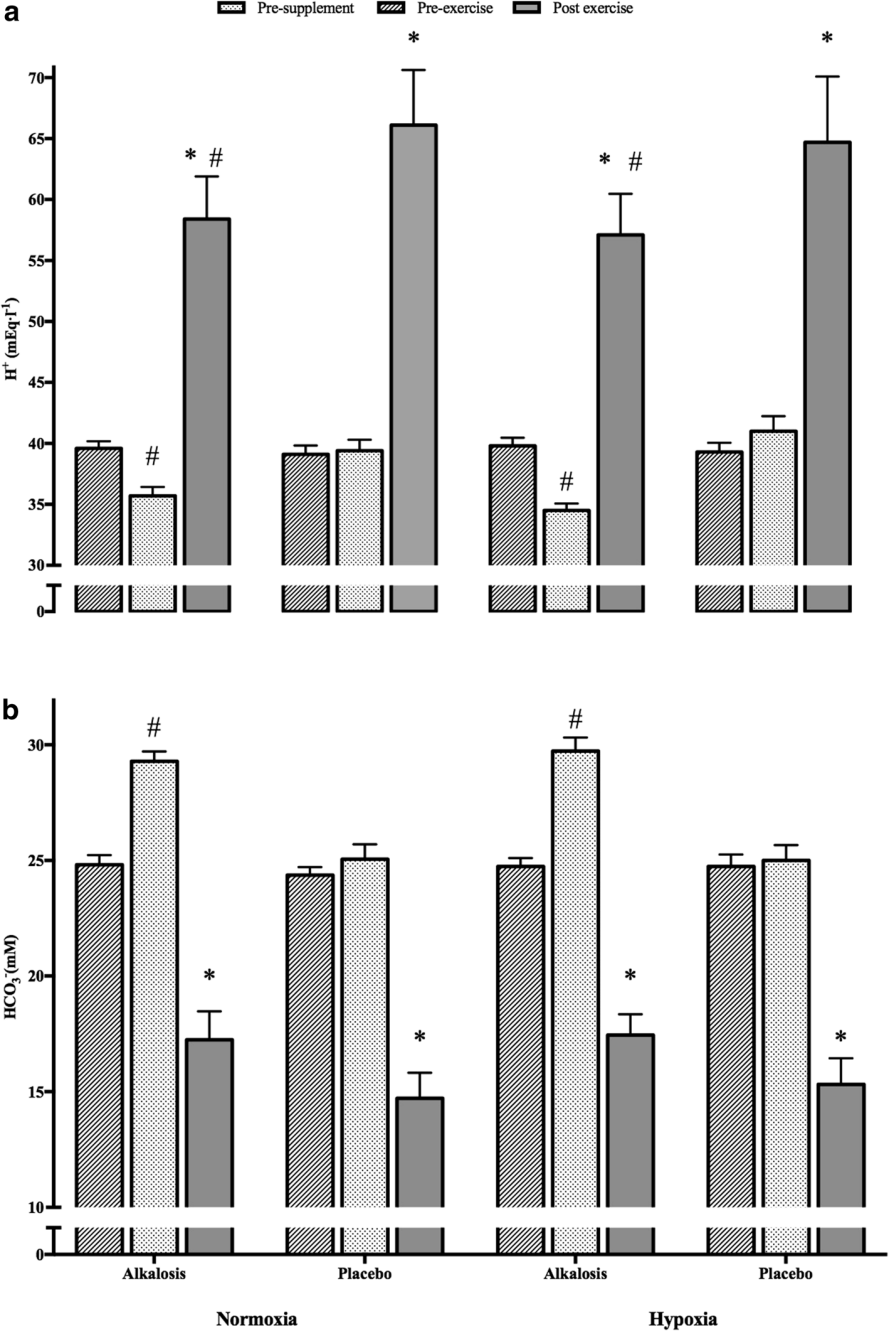
Graph **a** displays change in blood [H^+^] and graph (b) displays change in blood [HCO_3_^−^] across three time points during the four different experimental trials. *Error bars* are displayed as SEM. *Asterisk* represents significantly different from pre-supplement and pre-exercise time points (*p* < 0.05); *hash* represents significantly different from placebo conditions at the same time point

Pre-exercise alkalosis had a significant main effect on W’ (*p* < 0.001; ηp2 = 0.7) (Fig. 1) and TWD (*p* = *0.015;* ηp^2^ = 0.46) (Fig. 2). This represents a 14% increase in W’ under normoxic (*p* = 0.006) and an 18% increase under hypoxic (*p* = 0.001) conditions with NaHCO_3_ compared to placebo. Accordingly, magnitude based inferences determined a very likely effect under normoxia and most likely effected under hypoxia (Table 3). Pre-exercise alkalosis also elicited a positive effect on TWD, with post hoc comparisons showing a 5.5% (*p* = 0.048) and 4.8% (*p* = 0.01) increase under normoxic and hypoxic environments, respectively. In contrast, there was no supplement effect on CP (*p* = 0.41; ηp^2^ = 0.06). Critical power was, however, effected by the environmental conditions (*p* < 0.001; ηp^2^ = 0.8), with an overall mean reduction of 44.5 ± 23.2 W (Table 2). Similarly, an overall significant environmental effect on TWD was found (*p* < 0.001; ηp^2^ = 0.8) with hypoxia eliciting a mean 10.7% reduction. Conversely, W’ was not influenced by the environmental conditions (*p* = 0.59; ηp2 = 0.02). Comparison of CP to EP demonstrated an overall significant main effect across conditions (*p* = 0.004; ηp^2^ = 0.73), although pairwise comparisons were non-significant; while W and W’_EP_ did not differ (*p* = 0.210; ηp^2^ = 0.15). Pearson’s correlation between the effect of alkalosis reported a significant relationship between the effect on TWD and the effect on CP under normoxic (*r* = 0.92; *p* < 0.001) and hypoxic environments (*r* = 0.83; *p* = 0.001). Whereas, the relationship between the effect on TWD and W’ was not significantly different under normoxia (*r* = 0.52; *p* = 0.09) and hypoxia (*r* = 0.2; *p* = 0.54).

**Table 2:** Overview of 3-min all-out critical power test data

Variables	PLN	ALN	PLH	ALH
CP (W)	304.2 ± 40.0*	313.2 ± 48.5*	263.3 ± 34.3	264.8 ± 3
W’ (kJ)	15.1 ± 6.2^#^	17.4 ± 5.1	15.2 ± 4.9^#^	17.9 ± 5.2
EP (W)	308.2 ± 43.3	314.2 ± 55.5	267.0 ± 31.7	267.1 ± 29.4
W’EP (kJ)	14.1 ± 6.3	17.1 ± 5.0	14.5 ± 5.6	17.4 ± 5.2
TWD (kJ)	69.9 ± 10.2*^#^	73.8 ± 10.5*	62.6 ± 7.2^#^	65.6 ± 6.0
PPO (W)	697 ± 91	694 ± 87	694 ± 90	695 ± 86
Blood Lactate (mmol·l−1)	13.9 ± 5.2	15.5 ± 7.0	14.7 ± 6.4^#^	16.5 ± 5.4
HRpeak (bpm)	175.1 ± 7.1	175.3 ± 6.8	175.8 ± 7.4	176.1 ± 6.9
SpO_2_ (%)	94.0 ± 2.1*	93.7 ± 2.3*	83.5 ± 7.1	84.4 ± 3.9
RPE	18.8 ± 1.8	19.3 ± 1.6	19.0 ± 1.9	19.3 ± 1.6

Values reported as a mean ± SD

*PLN* represents the placebo normoxia condition, *ALN* represents the alkalosis normoxia condition, *PLH* represents the placebo hypoxia condition, *ALH* represents the alkalosis hypoxia condition. Blood lactate represents blood lactate accumulation during exercise

*Denotes significantly different to corresponding hypoxic trial (*P* < 0.001)

^#^Denotes significance to corresponding alkalosis trial (*p* < 0.001)

**Table 3:** Magnitude-based inference of performance effects from sodium bicarbonate supplementation compared to placebo at sea level and hypoxic environments

Environmental condition	Variable	Mean difference (90% confidence interval)	Non-clinical MBI
Sea level	TWD (kJ)	3.9 (± 3.1)	Very likely
	W’ (kJ)	2.3 (± 1.7)	Very likely
	CP (W)	9.0 (± 14.9)	Unclear
Hypoxia	TWD (kJ)	3.0 (± 1.2)	Most likely
	W’ (kJ)	2.8 (± 1.1)	Most likely
	CP (W)	1.4 (± 9.8)	Unclear

Non-clinical magnitude based inferences are interpreted with the criteria outlined in the “Statistical analysis” section

A significant two-way [supplement x time] interaction was detected on [HCO_3_^−^] (*p* < 0.001; ηp^2^ = 0.19) and [H^+^] (*p* < 0.001; ηp^2^ = 0.78), indicating NaHCO_3_ supplementation had a significant effect on blood [HCO_3_^−^] and [H^+^] (*p* < 0.001) as displayed in Fig. 2. A significant main effect with alkalosis was evident on change in [HCO^−^] during exercise (*p* < 0.001; ηp^2^ = 0.68), although the environmental effect was not significant (*p* = 0.615; ηp^2^ = 0.02). Further, pairwise comparisons revealed a 28% (*p* < 0.001) and 27% (*p* < 0.001) greater increase in [HCO^−^] reduction with pre-exercise alkalosis in the respective normoxic and hypoxic trials, compared to placebo (Fig. 2). Equivalently, blood [lactate] change during exercise was significantly increased with alkalosis by 10% under normoxia and 15% under hypoxia (Table 2) (*p* = 0.005; ηp^2^ = 0.54) but environmental conditions had no influence (*p* = 0.41; ηp^2^ = 0.06). Despite this, the significant supplement effect on change in [bla] only manifested during hypoxic conditions (mean difference =−2.22 nM; *p* = 0.012) but not during normoxic conditions (mean difference =−1.58 nM; *p* = 0.08). In contrast, change in [H^+^] from pre- to post-exercise did not change with neither a supplement (*p* = 0.82; ηp^2^ = 0.01) nor environment (*p* = 0.38; ηp^2^ = 0.07) effect (Fig. 2). Furthermore, mean values of PO_2_ and PCO_2_ across conditions are reported in Table 4.

**Table 4:** Blood PO_2_ and PCO_2_ during the CP experimental trials

Variables	PLN	ALN	PLH	ALH
Pre-supplement PCO2 (kPa)	5.3 ± 0.5	5.5 ± 0.5	5.1 ± 0.6	5.4 ± 0.6*
Pre-exercise PCO2 (kPa)	5.8 ± 0.3	5.7 ± 0.2	5.4 ± 0.4	5.7 ± 0.5
Post-exercise PCO2 (kPa)	5.2 ± 0.4	5.4 ± 0.5	5.1 ± 0.5	5.4 ± 0.5*
Pre-supplement PO2 (kPa)	9.0 ± 1.5	8.7 ± 1.3	8.3 ± 1.5	8.3 ± 2.0
Pre-exercise PO2 (kPa)	8.2 ± 1.3	8.6 ± 0.9	8.3 ± 1.7	8.5 ± 2.1
Post-exercise PO2 (kPa)	9.0 ± 3.7	10.0 ± 2.1	9.0 ± 2.0	7.6 ± 1.4

*PLN* represents the placebo normoxia condition, *ALN* represents the alkalosis normoxia condition, *PLH* represents the placebo hypoxia condition, *ALH* represents the alkalosis hypoxia condition

*Denote significant difference from placebo condition in the same environmental condition

## Discussion

This study was designed to determine the effect of NaHCO_3_ on the W’ parameter of power-duration relationship. The principle and novel finding was that attaining individualised peak blood alkalosis, appears to enhance the magnitude of W’ under both normoxic and hypoxic conditions. Furthermore, an increase in total work done across the 3 min exercise period was evident; along with greater reduction in blood [HCO^−^] and increased [bla] during exercise with pre-exercise NaHCO_3_ treatment. This investigation also revealed the magnitude of CP declines under moderate hypoxic exposure, supporting previous research (Dekerle et al. 2012; Simpson et al. 2015; Shearman et al. 2016). However, manipulation of acid–base status was deemed to have no effect on CP determination. Therefore, NaHCO_3_ may be an effective ergogenic aid by increasing work performed above CP and, also, the total work performed in the severe intensity domain.

The primary finding of the study is in contrast with previous research by Vanhatalo et al. (2010b), who, in a normoxic environment, reported no effect with NaHCO_3_ ingestion on the W’ parameter. The exercise protocols in both studies were matched, although the calculation of W’ did however differ, with W’ determined from the final 30 s power output by Vanhatalo et al. (2010b) whereas the present study used the lowest 30 s average power output. There were no significant differences in the current study, however, in W’ between the methods of calculation and therefore this is not likely to explain the differences. There are, however, two main methodological differences that may explain the opposing finding. Recent research has identified exogenous NaHCO_3_ ingestion is subject to inter-individual variance in blood pH and HCO_3_^−^ (Miller et al. 2016; Stannard et al. 2016). The current investigation accounted for this varied blood analyte response through individualised NaHCO_3_ administration prior to exercise, which ranged from 40 to 70 min and represented a significant increase in blood alkalinity and [HCO_3_^−^] prior to exercise (Fig. 2). The only previous investigation to employ this NaHCO_3_ administration protocol reported a larger variance between 10 to 90 min to peak alkalinity within in a recreationally active cohort (Miller et al. 2016). On this premise, it is reasonable to suggest that the standardised 60 min pre-exercise ingestion used by Vanhatalo et al. (2010a, b) may not have induced maximal peak alkalosis within all participants; thereby masking the influence of alkalosis on W’. The differing results with the current study could also be attributed to the training status of participants used, as the ergogenicity of NaHCO_3_ has been suggested to be greater in trained individuals (Carr et al. 2011; McNaughton et al. 2016). Alternatively, the differing results could be attributed to the recently identified inter-individual variability in performance response to NaHCO_3_ supplementation (Saunders et al. 2014a; Froio de Araujo Dias et al. 2015).

The magnitude based inference analysis determined the ergogenic effect of sodium bicarbonate on W’ to be ‘very likely’ and ‘most likely’ under normoxic and hypoxic conditions, respectively. The current investigation is the first to demonstrate a significant and meaningful improvement in performance from NaHCO_3_ under acute hypoxic conditions, in contrast with as previous investigations reporting no effect (Saunders et al. 2014b; Flinn et al. 2014). The conflicting results to previous investigations may be attributed to the intensity and type of exercise, which were supra-maximal and intermittent in both previous studies, or the training status of participants, who were recreationally trained. Furthermore, it should be acknowledged that acidosis is suggested not to be an important contributor to fatigue at hypoxia; indeed, maximal repeated sprint performance is shown to be independent of the presence of muscular acidosis following exhaustive exercise (Morales-Alamo et al. 2015). While other investigations have reported findings in direct contract by suggesting acidosis can better maintain performance at hypoxia (Schoene et al. 1983; Fulco et al. 2006). Therefore, importance of alkalosis on exercise performance cannot be concluded from the observations of the current study and both acidosis and alkalosis may be beneficial to performance though differing mechanism of action. Interestingly, the magnitude based inferences revealed a larger effect of NaHCO_3_ under hypoxia compared to normoxia, which may be due to the greater acidic stress and the subsequent increased reliance on the HCO_3_^−^ buffering system during exercise. However, 3 min CP protocol was not primarily designed as a sensitive measure to detect changes in overall performance but rather establish CP and W’. Therefore, the use of fixed intensity exercise tolerance protocol, such as the Cycling Capacity Test 110% (Saunders et al. 2014a), may provide a more appropriate high-intensity protocol to test the difference in efficacy of NaHCO_3_ between hypoxic and normoxic environments.

This study provides a unique mechanistic insight into the determinants of W’, which is subject to uncertainty within scientific literature (Poole et al. 2016), given the multi-faceted central and peripheral mechanisms of exercise tolerance and fatigue (Amet and Verkerke 2009). Using ^31^P magnetic resonance spectroscopy (MRS) muscle metabolite responses to exercise above CP have been characterised, with a rapid accumulation of H^+^ and P_i_ observed (Jones et al. 2008). Acidosis is associated with exercise above CP and is likely to, at least in part, contribute to exercise intolerance at intensities within the severe intensity domain. Moreover, alleviating acidosis is shown to result in increased muscle glycogen utilisation (Hollidge-Horvat et al. 1999; Percival et al. 2015), and given the association between glycolytic flux and W’ (Miura et al. 2000), it is conceivable that increased glycolysis with alkalosis may contribute to an increased W’. Pre-exercise NaHCO_3_ supplementation enables an increased H^+^ efflux from the intramuscular regions to the extracellular blood compartments (Roth and Brooks 1990), which corresponds to a reduction in the rate of intramuscular H^+^ accumulation during exercise (Stephens et al. 2002) and a concomitant increase in intramuscular glycogen utilisation (Percival et al. 2015). Therefore, the overall improvement in W’ and total work done in this study could be attributed to the blood acid–base biochemical changes during exercise. Indeed, a significantly greater reduction in blood [HCO^−^] during exercise with alkalosis is indicative of a superior HCO_3_^−^ buffering activity during exercise. Thereby, attenuating the rate of H^+^ accumulation and thus, actively delaying acidosis development. Furthermore, a significant increase in [bla] was evident following NaHCO_3_ treatment in comparison to placebo by 15% under hypoxic conditions and non-significant difference of 10% under normoxia. This enhanced lactate accumulation presents an indirect biomarker of upregulated glycolytic flux during exercise, which has been cited as the mechanism by which NaHCO_3_ is ergogenic (Hollidge-Hovart et al. 1999). This significant [bla] increase under hypoxia may also explain the greater magnitude of effect in hypoxia. Accordingly, it is proposed that a combination of enhanced intramuscular H^+^ efflux and increased glycolytic flux offers mechanistic explanations to the enhanced W’ and improved TWD with NaHCO_3_.

Nevertheless, this study did not directly quantify anaerobic energy contribution and therefore, the improved W’ and TWD cannot be directly attributed to an increased glycolytic flux. Despite lactate cited as an indirect marker of glycolysis, the increase observed could also be explained by a reduction in lactate uptake by inactive tissue (Granier et al. 1996) or an increased lactate efflux working intramuscular regions to extracellular space (Bishop et al. 2004) from the supplementation of NaHCO_3_. Furthermore, a recent investigation has suggested anaerobic exercise is not hindered with acidosis (Morales-Alamo et al. 2015) and, therefore, without assessment of appropriate glycolytic enzyme activity and muscle glycogen utilisation in this study, the increased anaerobic energy contribution can only be hypothesised. While it may be intuitive to identify W’ as an indicator of anaerobic energy supply, more recent definitions have suggested a relationship with VO_2_ kinetics (Poole et al. 2016). As such, alterations in O_2_ delivery and utilisation may also effect W’. Indeed, Nielsen et al. (2002) found an improved maintenance in SPO_2_ during a maximal 2000 m rowing with constant infusion of HCO_3_^−^ to maintain homeostatic pH during exercise; which the authors cited as a reason for the improved exercise performance. In the current study, end exercise SPO_2_ did not differ between conditions, however, this may be due to constant infusion of HCO_3_^−^ in the previous study compared to a single pre-exercise bolus in the current investigation. It is conceivable that differences in SPO_2_ response during exercise may have occurred in the current study. However, continuous measurement of SPO_2_ were not taken and therefore increased O_2_ delivery during exercise cannot be ruled out as a potential mechanism for the improved W’ and TWD.

An interesting observation in this study was the strong correlation between the effect of alkalosis on TWD and on CP under normoxia and hypoxia. Therefore, highlighting the substantial effect changes in CP can have on TWD, which may be explained as CP represented a larger proportion of intensities up to VO_2peak,_ in comparison to the severe intensity domain alone (Burnley and Jones 2016). However, no significant main effect of induced alkalosis on CP was noted, suggesting pre-exercise alkalotic inducement has no clear benefit on exercise performance at intensities at or below CP. This is expected, given a steady state of [H^+^] is maintained within 3 min of exercise at the CP intensity (Jones et al. 2008). Nonetheless, pre-exercise NaHCO_3_ supplementation has been demonstrated to improve exercise tolerance at CP by 23.5% (Mueller et al. 2013). Supporting research addressing the ergogenic effect of induced alkalosis on lower intensity exercise performance is scant; however, research does suggest alkalosis can improve performance up to 60 min (McNaughton et al. 1999). Despite the results of the current investigation, the ergogenic effect of longer duration activities at or below CP intensity cannot be dismissed; although potential ergogenic benefits are likely to more efficacious within W’ exercise intensity ranges. Equally, the likelihood of alkalosis to exhibit a negative impact on CP should not be dismissed. Alkalosis increases muscle glycogen utilisation (Percival et al. 2015), and due to the association between low muscle glycogen and a lower CP (Muira et al. 2000), it is appropriate to hypothesise NaHCO_3_ may diminish CP further during prolonged exercise.

The current study was designed to determine the effect of NaHCO_3_ on the W’ parameter of the power-duration relationship under normoxia and acute hypoxia. However, the use of a single acute hypoxic magnitude may limit the generisability of the results to alternative hypoxic doses. Indeed, medium term (3 days) severe altitude exposure (5050 m) is suggested to negatively impair both CP and W’ (Valli et al. 2011). Therefore, the response to NaHCO_3_ may be different under more severe hypoxic exposures and with a prolonged exposure. Nevertheless, the current study provides an insight into the NaHCO_3_ under an acute moderate hypoxic dose, common to environments athletes may train. A further limitation is that respiratory data were not collected during the 3 min CP test and therefore, the cardiopulmonary response to the environmental and supplemental interventions cannot be distinguished. Nevertheless, HR_peak_ and RPE did not differ between conditions suggesting exercise intensity was equivalent between conditions. It should also be highlighted that the low PO_2_ values were not reflective of an arterialised blood sample, suggesting our arterialisation technique was not effective. Nevertheless, blood gas parameters (e.g. pH and HCO_3_^−^) are suggested to unaltered between arterialised and non-arterialised blood samples (Stannard et al. 2016) and therefore, the results remain valid; while also remaining consentient with previous scientific literature (Flinn et al. 2014; Saunders et al. 2014a, b; Froio de Araujo Dias et al. 2015; Miller et al. 2016) Furthermore, it is suggested future investigations should consider obtaining muscle biopsy and arteriovenous balance analysis to understand the metabolic response to NaHCO_3_ ingestion on W’.

## Conclusion

This study is the first to demonstrate that pre-exercise alkalosis has a beneficial effect on W’ under normoxic and hypoxic conditions. This was accompanied by a greater blood [HCO^−^] reduction and an increased [bla] during exercise with alkalosis, which present indirect markers of HCO_3_^−^ buffering activity and glycolytic flux. Taken together, it suggests W’ may be enhanced through improved regulation of acid–base status during exercise, which may mediate an increase in anaerobic energy contribution. Furthermore, this study also demonstrated that individualised NaHCO_3_ ingestion improved TWD during the 3 min exercise test, whilst also being the first investigation to report the ergogenic effects of NaHCO_3_ under acute hypoxia.

## Abbreviations

[HCO_3_^−^]: Bicarbonate ion concentration
CP: Critical power
FiO_2_: Fractional inspired oxygen
VT1: Ventilatory threshold 1
[H^+^]: Hydrogen ion concentration
O_2_: Oxygen
SPO_2_: Oxygen saturation
VO_2peak_: Peak oxygen uptake
PO_peak_: Peak power output
PCr: Phosphocreatine
NaHCO_3_: Sodium bicarbonate
NaCl: Sodium chloride
TWD: Total work done
kJ: Kilojoules
